# Correction to “Safety and Efficacy of Radiotherapy Combined With Sintilimab in Advanced NSCLC Patients Who Progressed on First or Second Line Therapy: A Prospective, Multiple Center, and Single‐Arm Study”

**DOI:** 10.1111/1759-7714.70066

**Published:** 2025-04-20

X. Feng, X. Liu, H. Guan, et al., “Safety and Efficacy of Radiotherapy Combined With Sintilimab in Advanced NSCLC Patients Who Progressed on First or Second Line Therapy: A Prospective, Multiple Center, and Single‐Arm Study,” *Thoracic Cancer* 16 (2025): e70043.

Figure 1 is incorrect due to the grouping of label errors.

Below is the correct Figure 1.

**FIGURE 1:**
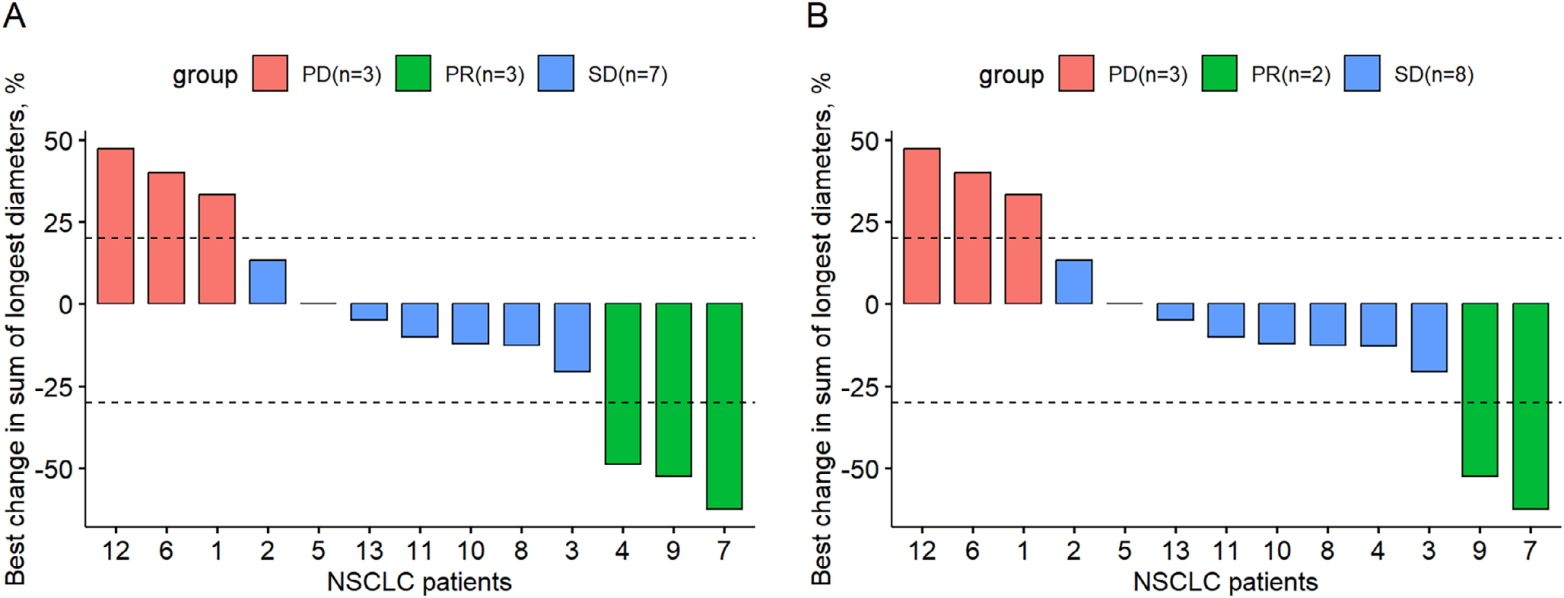
Waterfall plots of best response in individual patients. (A) Illustrated the best response of all evaluable patients, including those with lesions within the radiation field. (B) Presented the best response when assessing lesions excluding the radiation field. One patient who did not undergo radiological scans after disease progression was therefore not included in the waterfall plot. NSCLC, non‐small cell lung cancer; PD, progressive disease; PR, partial response; SD, stable disease.

We apologize for this error.

